# Golden Ager Chyawanprash with Meager Evidential Base from Human Clinical Trials

**DOI:** 10.1155/2022/9106415

**Published:** 2022-05-16

**Authors:** Rohit Sharma, Pradnya Kakodkar, Atul Kabra, Pradeep Kumar Prajapati

**Affiliations:** ^1^Department of Rasashastra and Bhaishajya Kalpana, Faculty of Ayurveda, Institute of Medical Sciences, Banaras Hindu University, Varanasi 221005, Uttar Pradesh, India; ^2^Department of Research, Dr D Y Patil Vidyapeeth, Pimpri, Pune, Maharashtra 411 018, India; ^3^University Institute of Pharma Sciences, Chandigarh University, Gharuan, Mohali, Punjab, India; ^4^Department of Rasashastra and Bhaishajya Kalpana, All India Institute of Ayurveda, New Delhi 110076, India

## Abstract

Chyawanprash (CP) is one of the most revered, effective, and best-selling health supplements of Indian Ayurvedic system of medicine, which is high on the trust and health quotient among Indian consumers. CP is an ancient synergistic blend of around fifty (50) medicinal herbs, herbal extracts with jam-like consistency, traditionally used to improve respiratory health, strengthen the immune system, prevent recurrence of infections, and over the decades is known for its anti-inflammatory and antioxidant properties. With the recent growth of commercialization, this traditional preparation is gaining popularity across the world. Various pharma firms are engaged in its commercial production, and this recipe is easily available on market shelves, with a common brand name “Chyawanprash.” The consumers generally pop up with some genuine queries or doubts regarding its usage and benefits. Though CP is well-trusted traditional preparation, however, limited scientific literature is available on understanding its clinical role. This critical review discusses the general consumer queries apropos its usage, critically analyzing the reported clinical studies, perspectives, and unmapped areas for future research.

## 1. Introduction

Ayurveda is a well approved treatment modality by the World Health Organization [1]. Chyawanprash (CP) is one of the most admired and therapeutically effective Ayurveda supplements widely used in Indian subcontinent. The CP recipe includes around fifty (some times more depending on the brand) bioactive herbs (with the major ingredient, *Amla* or Indian gooseberry) and herbal extracts, which are processed by the traditional Ayurvedic pharmaceutical process, and the final form of CP has the jam-like consistency. The composition includes several bioactive antioxidant, anti-inflammatory, adaptogenic, cytoprotective, and rejuvenating herbs with wide spectrum therapeutic potential. This preparation is well-trusted among Indian consumers and is traditionally used to prevent the recurrence of diseases, boost immunity, and to strengthen respiratory as well as overall health. The formulation is also included in authoritative literature, i.e., Ayurvedic Pharmacopoeia of India [2].

**Figure 1:**
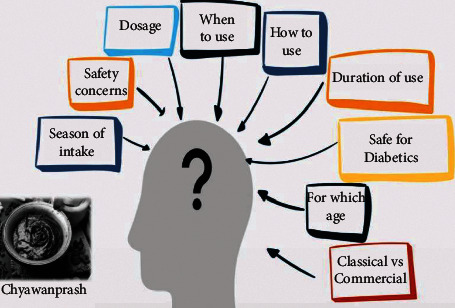
General doubts among consumers regarding CP usage.

With the commercialization trends, this ancient ayurvedic traditional recipe has also reached other parts of the world. It came across several opinions on the power-insight of this bioactive supplement that has had experiences of multiple Indian generations consuming this nutraceutical potion. As the electronic and print media and now-a-days over-the-top (OTT) media space has enhanced the reach of CP brands to the new age digital audience, the consumers pop several genuine queries (Figure 1), and the researchers and Ayurveda scholars owe them an answer for the same with substantial evidence. However, in scientific literature, which means human clinical trials undertaking CP research, the results are very meager. This study explores all the available clinical studies conducted using CP, critically assess its benefits, problems, and unexplored areas for future research.

## 2. Effect on Diabetes and Lipid Profile

Due to presence of sugar, honey, and cow ghee contents, CP is generally considered to be contraindicated in diabetes or lipid disorders. However, three clinical studies can be retrieved specially studying diabetes and CP. In one study, CP was found to reduce postprandial hyperglycemia in the oral glucose tolerance test and significantly reduced blood cholesterol levels as compared to the vitamin C control group [3]. One study evaluated the effects of CP in elderly people and found a decrease in cholesterol, triglycerides, and LDL (low-density lipoprotein) and increase in HDL (high-density lipoprotein) levels, which suggest its hypolipidemic role and validate its indications in geriatrics as already mentioned in Ayurveda classics [4]. In another clinical study on type-2 diabetics, the consumption of CP resulted in an insignificant change in HbA1c and blood sugar levels, which indicates its safe usage in mild to moderate diabetes controlled by oral hypoglycemic agents [5]. However, these studies seem to be not free from bias and the details are given in Table 1. Due to the scarcity of evidences, further in vivo and high-quality clinical investigations are required to understand the safety of CP in diabetes and reach a definite conclusion.

## 3. Suitability in Different Seasons

It is a general belief that CP is to be consumed in the winter season and many people are skeptic on whether it can be taken in summer or for the whole year. Thorough understanding of nature and properties of its ingredients suggest that it could be used in all seasons because the composition balances all three doshas or tridosha (body humours, viz., vata (kinetic), pitta (metabolic), and kapha (potential)) and helps to maintain the health. The tridosha work in harmony and regulate each other to maintain homeostasis, equilibrium, healthy body and mind, and any imbalance in tridosha leads to various health disorders [13]. Compelling evidences suggest that CP possess rich nutraceutical value along with potential antioxidant, anti-inflammatory, and immunomodulatory properties [14]; hence, its daily intake strengthens the immune system and protects all the yearlong. In a clinical study conducted on the 15–75 years age group (with no underlying organic disease), CP was found to reduce the disease symptoms of seasonal influences, improved pulmonary functions, quality of life, and provided 3-times immunity with no adverse effect(s) when used throughout the year [6].

Furthermore, Ayurveda doctrines state that summer is dominated by pitta dosha and thus related disorders are more prevalent in this season. CP contains a number of pitta-balancing ingredients viz. *Emblica officinalis* (*Amla* or Indian gooseberry), the prime ingredient of CP, *Pterocarpus santalinus, Asparagus racemosus, Elettaria cardamomum, Terminalia chebula, Nelumbium speciosum*, *Tribulus terrestris*, and ghee of Indian cow (*Bos indicus*) that help to maintain balance and stability pitta in the summer months.

The change in weather has direct impact on human health. Ayurveda believe that during the interseasonal period/the transition or junction between seasons (ritusandhi), the tridosha gets imbalanced, along with impaired digestion, immunity, and mental and physical strength, making the body more susceptible to diseases [15]. This seasonal change impact on immunity and human physiology is well-supported by recent reports [16, 17]. During these circannual seasonal changes, Ayurveda advocate optimally timed biopurifications and seasonal eating regimens with all measures to protect and strengthen immunity. Therefore, CP in regulated dosages (as digestion is weaker in ritusandhi) could be an advisable supplement to supercharge the immune-defense during ritusandhi. Beneficial effects of CP have also been reported in nasal allergies, viral infections, and seasonal influences [2, 18]. CP contains several weather friendly ingredients, and no any conspicuous report on the adverse effect is available on its usage in summers or during any specific season [2, 19, 20], which signifies that CP may be consumed yearlong (in any season) to enhance the body's immunity and reduce vulnerability to diseases. However, more supporting multicentric clinical studies with yearlong assessment are required for better understanding and to probe the adverse effects, if any.

## 4. Age, Dosage, and Duration Considerations in CP Usage

Ayurveda literature advocates the administration of CP as per the digestive capacity and strength of the recipient, so that it may not interfere with hunger and appetite for food. Ideally, it should be taken empty stomach and not to be mixed with any other food. In Ayurvedic Pharmacopoeia of India, the standard dose of CP is mentioned 12 g twice a day [21]. CP is designed in such a way that it is generally considered suitable for nearly all age groups above 3 years with appropriate dosage, generally 12–28 g per day, with lukewarm milk or water. Till now, CP has been clinically studied in diverse population in various therapeutic targets, as a preventive as well as curative preparation and as an adjunct/add on therapy or monotherapy. In animal toxicity studies (acute-dose oral toxicity, 28 days repeated-dose oral toxicity studies, and 90 days repeated-dose oral toxicity studies), CP was found to be safe in the recommended dosage for the defined period of time as no toxicity or bodyweight loss was found with all animals surviving throughout the study duration [7].

## 5. Critical Evaluation of the Clinical Studies around CP

Although CP was clinically evaluated in several studies (Table 1) in different age groups (ranging 5–75 years), various dosages (ranging 12–30 g per day), and different durations (from 28 days to 2 years), none of the study reported any conspicuous adverse reactions or specific safety concerns. Nevertheless, case studies should be encouraged to investigate if any adverse effects are found on its usage, e.g., in acute illness, exacerbation of chronic kidney disease or any organ/system-specific disorder, gastric irritation or any specific gastrointestinal condition, and individual intolerance to the components, so that the warning and contraindications, if any, can be highlighted. Table 1 provides the summary of different clinical studies conducted using CP. However, the major concern is that every study has used different amounts of CP for consumption and also varied frequency. There is no research till date, which has standardized the consumption amount and the frequency of intake. Careful evaluation of clinical reports reveals that the major factors in these studies that are indicative of bias are the poor study design, small sample size, use of commercial CP product for evaluation, and conflict of interest arising due to the authorship of the study by the employees of the company whose product is tested in the study (Table 1).

CP is a Rasayana preparation (tonic drug for rejuvenation and prevention of aging or diseases), and Ayurveda consider that the usage of Rasayana for longer durations is safe in stated therapeutic dosages [19]. Though to provide scientific basis, more evidences on such traditional formulations need to be generated. Well-stratified, multicenter, randomized controlled clinical trials of longer durations in diverse population, comparing the efficacy with placebo/standard groups with interim or longer follow-up, are warranted to establish the standard/defined dose and duration suitability as per different age groups (children, adults, and elderly). It should be investigated in different age groups that which adjuvants/vehicle/medium (among water, milk, or any other drink) is therapeutically safe and effective or whether CP should be taken alone. The safety studies are also required on CP usage during pregnancy and lactation for clearer understanding.

Though, scientifically, there is not enough and strong evidence to elucidate the benefits and mechanistic role of CP, more studies are warranted on this time-tested and highly effective formulation of the traditional Ayurveda system of medicine.

## 6. Commercialization and CP

Current science is witnessing continuous rational efforts of researchers validating the ancient wisdom on scientific grounds, commercialization, technological developments, skeptic, and more evidence-based approach toward any market product and drug development or product development to meet the needs of consumers [22–24]. In India, CP is on high demand since ages owing to its multiple health benefits, cultural linkage, endorsement by ancient visionary sages, and strong trust of general consumers on this ancient elixir to be safe and effective to all as a family tonic. Yet there lacks uniformity among the market samples of CP and its reasons are purely commercial. In the past decade, the pharmaceutical sector tried to modify the classical CP recipe as it keeps on (I) adding some extra ingredients (e.g., silver and gold) or slightly changing the composition, aiming to add on to the nutraceutical value or for specific therapeutic targets, (II) adding flavors/fruit-flavored variants (e.g., mixed fruit, orange, or mango flavor), and varieties of it, and (III) changing the forms of classical CP (e.g., making simple granules, cookies, sugar-free biscuits, chocolate granules, sugar-free CP, and replacing sugar with jaggery) with the motto to make the traditional brand appealing, attract the consumers, or meet the demands of every age group. If the products like CP (recipe or process) are prepared in noncompliance with ancient Ayurvedic literature or authoritative texts listed in the regulations and modifying it as a part of product development, the preparation will not remain classical, and it becomes a “proprietary product.” The ingredients and the preparation vary from company to company; however, the firms use the same traditional name in their product, perhaps to encash the trusted goodwill of name “Chyawanprash.” Nonetheless, the real meaning and original efficacy of CP should not be compromised. The transformation or development of any product must be well-tested on the safety and efficacy grounds; especially when an ancient time-tested classical formulation is modified, the claims of its variants should be validated with the proper support of in vitro, in vivo, or clinical investigations published in quality research journals [25, 26]. Despite several commercialization trends, CP is widely used nutraceutical supplement among Indian consumers which is evident from its huge market demand, media advertisements, visibility on market shelves, different brand variants, and multicultural or multiregional acceptability.

Furthermore, in the composition of commercially available CP, substitute herbs are being used in place of Ashtavarga (eight rare herbal ingredients), owing to its nonavailability, poor identification/authentication, and lack of chemical markers [27]. Identification, conservation, and cultivation efforts should be encouraged for these endangered Ashtavarga herbs, which is a shared responsibility of commercial firms, herbal drug regulatory authorities, and the concerned ministry, and holding hand together with agricultural departments and ethnomedicinal/traditional or folklore healers could also be of great help [28, 29].

CP is a complex mixture of dozens of bioactive phytochemicals with multitargeted biological effects; there lacks the adequate knowledge in understanding of its mechanistic role. There is also possibility of synergistic or antagonistic interactions between various compounds, and the cumulative effect of the combination may result in augmentation or nullification of individual effects [30, 31]. A wide area is open for the researchers to work upon.

## 7. COVID-19 and CP Usage

The popularity of CP is also evident from the fact that, amid this challenging coronavirus (COVID-19/SARS-CoV-2) pandemic period in India, there has been a rising awareness among the consumers to safeguard the health and boost the immunity, resulting in high demand of traditional supplements like CP, and the Indian Pharma sector witnessed a sharp exponential surge in CP sales. Also, the Ministry of AYUSH, Government of India, has recommended having 10 g CP in the morning as an immunity booster, which is likely to further drive market demand of this supplement. Two recent clinical trials investigated the role of CP in COVID-19 and showed its promising role in prophylaxis and managing mild to moderate symptoms [11, 32]. As the evidence exploring the preventative as well as curative role of CP to improve the quality of life in COVID-19 infected subjects on various health parameters is scarce, further clinical exploration is warranted on the emerging viral strains to substantiate the preliminary findings.

## 8. Conclusion and Future Perspectives

It is high time to investigate the CP like traditional products which have immense potential and offer safe and effective remedial options where the synthetic drugs have limited roles. In the present report, an attempt was made to understand the strengths and weaknesses of available clinical studies on CP. Furthermore, multicentric, randomized controlled clinical trials of longer durations are required, comparing with the placebo/standard groups. Long follow-ups are also needed to get a clearer understanding of its efficacy and safety. This report also discusses the manufacturing trends at the pharmaceutical industries level and lack of uniform preparation steps as well as the composition of this recipe. To manage this situation and to prepare quality formulation of uniform optimum standards, stringent steps are required at the level of drug regulatory authorities and policy makers. Consumer awareness on its safe and effective usage in prescribed dosage is also equally important. Any modification in classical preparation should be tested well on safety and efficacy grounds prior commercialization and should be treated as proprietary herbal product, not labelling it as classical Ayurveda preparation. The present report is expected to clear certain misperceptions regarding CP usage and serve as guiding tool to researchers working in the area of bioactive natural products, drug development, functional foods, and nutraceuticals. It is recommended that country wide experts may be invited to discuss and formulate standardized study design for CP research.

## Figures and Tables

**Table 1:** Critical analysis of the scientific clinical studies around CP.

Author	Sample size and (age of the participants)	Dose and duration	Key effects of CP consumption	Critical findings	Reference
Manjunatha S et al.	*N* = 5. Healthy adult male volunteers (20–32 years)	16 weeks (first 8 weeks intervention was given and next 8 weeks no supplements)	Antihyperglycemic and hypolipidemic effects	The sample size is very small. During the no supplement 8-week period, no significant effects were recorded.	[3]
Dalai SK et al.	*N* = 30. Healthy elderly volunteers (40–70 years)	24 g/day for 40 days.	Antiatherogenic, hypolipidemic, and improves strength	The conclusive results are based on the assessment carried out on only 9 participants. CP as per Charaka Samhita reference was the most efficient	[4]
Kumar S et al.	*N* = 121. Type-2 diabetes subjects (18–70 years).	One teaspoonful, twice daily for 90 days	Statistically insignificant change in HbA1c, blood sugar levels, lipid profile, and liver and renal function tests	Error in age selection criteria, as in younger ages, one can find minimal cases of type II diabetes. This study is about proprietary product Chyawanprash which contains sugar substitute	[5]
Sastry JLN et al.	*N* = 40. Subjects having the history of allergy and respiratory illnesses (5–75 years)	12 g, twice a day for 12 weeks.	Beneficial in nasal allergies and viral respiratory infections	Conflict of interest. The author of the study is employed at the company whose product is evaluated in the study.	[6]
Sastry JLN et al	*N* = 177. Subjects with no underlying organic disease (15–75 years)	12 g, twice a day for 2 years.	Reduction in disease symptoms due to seasonal influences, improved respiratory functions, quality of life and immunity	It is a robust study. Conflict of interest that the author of the study is employed at the company whose product is evaluated in the study is not mentioned.	[7]
Gupta a et al.	*N* = 313. School going healthy children (5–12 years)	6 g, followed by a cup of milk, twice a day for 6 months.	More than 2 times protection from immunity-related illness (episodes of infections or allergic disorders) and improves fitness, strength, stamina, and quality of life.	A robust clinical study. A conflict of interest is mentioned. The author of the study is employed at the company whose product is evaluated in the study.	[8]
Yadav JS et al.	*N* = 25. Male smokers	20 g, twice a day for 2 months.	Genoprotective	Efficiently conducted clinical study.	[9]
Debnath PK et al.	*N* = 99. Pulmonary tuberculosis patients (10–65 years)	10 g thrice daily (total 30 g/day), used as adjunct therapy with antitubercular drugs for 28 days.	Toxicity reduction and early restoration by increasing the bioavailability of antitubercular drugs	Conclusive results are based on findings of only 15 patients. The sampling and study design is complex and lacks clarity.	[10]
Sanger NS et al.	*N* = 30. Confirmed COVID-19 mild to very mild symptoms (18–75 years)	12–24 g, with water or milk for 28 days.	Significant improvement in immunity and respiratory clinical symptoms	A robust clinical study.	[11]
Kumar SS et al.	*N* = 75. Healthy college students (mean age of 19.63 ± 1.55 years)	15 g, twice a day for 150 days.	Improvement in cognitive functions	Methodologically performed clinical study.	[12]

## Data Availability

The data used to support the findings of this study are available from the corresponding authors upon request.
